# Supplementary material for: Systematic review of post-COVID condition in Nordic population-based registry studies
Source: Nat Commun. 2025 Jul 1;16:5717. doi: 10.1038/s41467-025-60784-4 (PMC12219526; doi:10.1038/s41467-025-60784-4)
Supplement: Supplementary file 1 — Supplementary Information [file 41467_2025_60784_MOESM1_ESM.pdf]

Additional information

Supplementary Table 1.

Author	Country	Reason for exclusion
**Abzhandadze (2023)**	Sweden	Overlap with newer publication
**Arendt-Nielsen (2023)**	Denmark	No non-covid controls
**Arntzen (2022)**	Norway	Follow-up ≤ 90 days
**Gulseth (2022)**	Norway	Follow-up ≤ 90 days
**Grosland (2023)**	Norway	Aggregated data
**Haase (2021)**	Denmark	Follow-up ≤ 90 days
**Hagglof (2023)**	Sweden	Different outcome
**Hedberg (2023)**	Sweden	Overlap with newer publication
**Jakobsen (2023)**	Denmark	Different study design
**Kildegaard (2022)**	Denmark	Only abstract available
**Kisiel (2021)**	Sweden	Different population
**Liliequist (2023)**	Sweden	Only abstract available
**Methi (2022)**	Norway	No non-covid controls
**Moestrup (2023)**	Denmark	Follow-up ≤ 90 days
**Norgard (2023)**	Denmark	Different population
**O'Regan (2023)**	Denmark	Pre-print
**O'Regan (2023)**	Denmark	Different study design
**Palstam (2021)**	Sweden	Overlap with newer publication
**Ritsinger (2023)**	Sweden	Different outcome
**Tholin (2023)**	Norway	No non-covid controls
**Westerlind**	Sweden	Overlap with newer publication
**Wettermark (2022)**	Sweden	Only abstract available
**Zettersten (2021)**	Sweden	Different outcome

Supplementary Table 2.

Detailed overview of how authors defined their outcomes, populations, and controls.

Author	Definition of PCC/ PCC associated sick leave/ New onset disease and control group
**Abzhandadze (2024) (27)**	Outcome: The outcome variable measured was the number of days on sick leave due to COVID-19, as recorded by the Swedish Social Insurance Agency (SSIA). To be included in the follow-up, the sick leave had to contain at least one instance attributed to COVID-19, confirmed through laboratory testing and coded with ICD U07.1. In cases where the virus was not identified, ICD code U07.2 was used instead. Sick leave episodes could also encompass other diagnoses, provided the interval between them was less than two weeks and the additional diagnoses were considered related to COVID-19. These related conditions could include viral infections, fever, or a subsequent period of sick leave again attributed to COVID-19.<br>Population: The study population included individuals aged 18 years or older residing in Sweden who received sickness benefits for COVID-19, as identified by ICD codes U07.1 or U07.2. Eligible sick leave episodes had to begin between March 1 and August 31, 2020.
**Andersson (2024) (37)**	Outcome: The primary outcome was hospitalization due to any infectious disease. Secondary outcomes were defined by categorizing the infectious disease diagnoses into specific subtypes: upper respiratory tract infections, lower respiratory tract infections (excluding influenza), influenza, gastrointestinal infections, skin infections, urinary tract infections, selected invasive bacterial infections (including sepsis, meningitis, endocarditis, and osteomyelitis), as well as other infectious disease types.<br>Population: The study cohort was assembled to reflect the general Danish population born in 1972 or earlier - corresponding to individuals turning 50 or older in 2022. Inclusion criteria required Danish residency at baseline and no prior SARS-CoV-2 infection before entering the study. A SARS-CoV-2 infection was identified through a registered positive PCR test and was treated as a time-varying exposure in the analysis.
**Bygdell (2023a) (36)**	Outcome: Post-COVID condition (PCC) was identified by the presence of the ICD-10 code U09.9, either as a primary or secondary diagnosis, in one of the national health registers (NPR, VEGA, or VAL). The PCC index date was defined as the first occurrence of this code more than 28 days after the individual’s COVID-19 index date.<br>Population:Individuals were considered COVID-19 cases if they had an ICD-10 diagnosis of COVID-19 (U07.1 or U07.2), listed as either a primary or secondary diagnosis in the NPR, VEGA, or VAL registers, and/or a recorded positive SARS-CoV-2 PCR test result in SmiNet. The COVID-19 index date was defined as the earliest of these events, using the hospital admission date for inpatient diagnoses in the NPR.
**Bygdell (2023b) (35)**	Outcome: Post-COVID condition (PCC) was identified using the ICD-10 diagnosis code U09.9.<br>Population: The study included all children aged 6 to 17 years living in Sweden’s two largest regions. Data on inpatient and outpatient

	visits—both from specialists and primary care providers—were obtained from high-quality national and regional health registers with near-complete coverage (as detailed in eMethods in Supplement 1). Eligible participants had a confirmed COVID-19 infection between January 31, 2020, and February 9, 2022.
**Gronkjaer (2023) (38)**	Outcome: Neurological sequelae of COVID-19 were defined as any first-time diagnosis of a neurological disorder, based on specific ICD-10 codes (A066, A17, A321, A390, A521–A523, A80–A89, B003–B004, B010–B011, B020–B021, B050–B051, B060, B261–B262, B375, B451, B582, E236A, F00–F03, G00–G99, I60–I69, M35.0, M32, M05–M06, M08.0), recorded from inpatient, outpatient, or emergency room encounters. The onset of illness was marked by the date of the first hospital contact. Diagnoses that were only suspected or not confirmed were excluded.<br>Secondary outcomes consisted of the first diagnosis within specific neurological categories: Parkinson's disease and parkinsonism, neurodegenerative diseases, any type of dementia (including Alzheimer's disease and vascular dementia), immune-mediated conditions such as multiple sclerosis and Guillain–Barré syndrome, other immune-mediated disorders, epilepsy, headache, narcolepsy, disorders of nerves, nerve roots and plexuses, polyneuropathy, neuromuscular diseases, myopathies, other neuromuscular conditions, cerebrovascular diseases, infections of the central nervous system (CNS), viral CNS infections, and other neurological disorders.<br>Population: The study population comprised all individuals in Denmark who underwent SARS-CoV-2 testing—both those with negative and positive results—over nearly two years from the beginning of the pandemic. A confirmed COVID-19 case was defined by a positive PCR test for SARS-CoV-2 from either a nasopharyngeal or tracheal sample.
**Hedberg (2024) (17)**	Outcome: Post-COVID condition (PCC) was defined as receiving the ICD-10 code U09.9 in primary care, outpatient specialist care, or inpatient care within 90 to 240 days following the first positive SARS-CoV-2 test.<br>Population: The study included individuals born in 2018 or earlier who had a PCR-confirmed primary SARS-CoV-2 infection between October 1, 2020, and February 8, 2022—a time when public testing was available and the PCC diagnosis was in use. To allow for accurate assessment of baseline characteristics, only those who had resided in Stockholm County since January 31, 2019 (one year prior to Sweden's first confirmed COVID-19 case), were included.
**Hetlevik (2023) (22)**	Outcome: The outcomes included 18 symptoms frequently associated with post-COVID-19 condition, as recorded by general practitioners.<br>Population: The exposed group consisted of all individuals who had a positive PCR test for SARS-CoV-2 between February 21, 2020, and February 20, 2021. A comparison group of uninfected individuals—those without a recorded positive PCR test—was selected using exposure density sampling.
**Jacobsen (2022) (11)**	Outcome (COVID-19 cohort inclusion/exclusion criteria): The primary analysis included all individuals who tested positive for COVID-19 via PCR between January 1 and May 30, 2020, with inclusion based on the date of the first positive test. Individuals younger than 18 or older than 64 years were excluded, as were

	those not considered part of the workforce (e.g., recipients of early retirement; see Table S1). In addition, individuals who died or emigrated within 30 days of the inclusion date were also excluded.<br>Population (comparison group): For comparative purposes, patients hospitalized with influenza between February 1, 2019, and May 30, 2020, were included. This time frame differed from that of the COVID-19 group to ensure an adequate number of influenza cases were captured.
**Kildegaard (2022) (21)**	Outcomes: During the intermediate phase, the study assessed the first recorded inpatient or outpatient hospital diagnosis of conditions potentially linked to SARS-CoV-2 infection. These included venous thromboembolism, multisystem inflammatory syndrome in children (MIS-C), myocarditis, pneumonia, encephalitis, Guillain-Barré syndrome, and other neuroimmune disorders. In the post-acute phase, outcomes were defined as the initiation of medications that may indicate complications or lingering symptoms of COVID-19 that did not necessarily result in hospitalization. These included short-acting β2 agonists, inhaled corticosteroids, paracetamol (acetaminophen), non-steroidal anti-inflammatory drugs (NSAIDs), and antibiotics typically used in Denmark for respiratory tract infections.<br>Population: The population included children and adolescents who tested positive for SARS-CoV-2 and were subsequently assigned a diagnosis code indicating persistent symptoms related to the infection (i.e., long COVID).
**Lund (2021) (20)**	Outcomes:The study examined a range of post-acute health effects, including delayed complications, chronic illnesses, persistent symptoms, and patterns of prescription drug use—focusing on outcomes potentially associated with SARS-CoV-2 beyond the initial infection period. Health care utilization was also assessed by calculating event rates per 1,000 individuals for general practitioner consultations, outpatient hospital visits, emergency department visits, and hospital admissions. These were measured across two timeframes: from 6 months to 2 weeks before the SARS-CoV-2 test, and from 2 weeks to 6 months after. Multiple visits by the same individual were counted, and data on GP visits were drawn from the Danish National Health Insurance Register.<br>Population: The study included all individuals in Denmark who had a positive or negative RT-PCR test for SARS-CoV-2 between February 27 and May 31, 2020. Exclusion criteria were less than one year of residency in Denmark, inconclusive test results, or death occurring within two weeks of the test date.
**Magnusson (2022) (23)**	Outcome: The study assessed overall health care utilization, drawing on data from primary care consultations recorded in the Norway Control and Payment of Health Reimbursement (KUHR) database and specialist care encounters from the Norwegian Patient Register.<br>Population: The exposed group comprised individuals with one or more confirmed positive PCR tests for SARS-CoV-2, based on the date of their first positive result. These participants had not been hospitalized with COVID-19, although a supplementary analysis also considered those who had been hospitalized.<br>Two comparison groups were used: Negative test group – individuals with one or more negative PCR tests and no prior positive result. For participants with multiple negative tests, one test

	date was randomly selected to balance health care use patterns before and after testing.<br>Untested group – individuals with no recorded PCR test results, who were randomly assigned a hypothetical test date to serve as a reference group.
**Magnusson (2022) (24)**	Outcome: The study examined commonly reported post-COVID-19 symptoms identified in systematic reviews, as documented in medical records with high validity and reliability. These outcomes were recorded from 14 days after a positive test onward and included musculoskeletal pain, fatigue, cough, heart palpitations, shortness of breath, anxiety or depression, and brain fog, as well as the presence of any of these symptoms. Individuals could have multiple complaints, and no outcome was assumed to exclude the occurrence of another (e.g., a record of fatigue did not preclude a record of cough).<br>Population: The study population consisted of residents who tested negative for SARS-CoV-2 during the period in Norway when the Omicron and Delta variants were both circulating at significant levels, and the variant type was known.
**Magnusson (2023) (25)**	Outcome: The study investigated medical symptoms and complaints documented in primary care and emergency services by general practitioners and physicians. These were categorized using ICPC-2 codes into three main symptom groups: Pulmonary symptoms: shortness of breath or dyspnea (R02), and cough (R05); Neurological symptoms: impaired concentration, memory problems, or brain fog (P20); General symptoms: fatigue (A04, A05, A29)<br>Population: The comparison population included two groups:<br>1. Test-negative individuals: persons with one or more negative SARS-CoV-2 test results during the inclusion period. If multiple negative tests existed (inside or outside the inclusion period), one was randomly selected. Those whose selected test date fell outside the inclusion window were excluded to ensure equal inclusion chances regardless of testing frequency. 2. Untested individuals: those who were never tested for SARS-CoV-2 at any point. Each was randomly assigned a hypothetical test date within the inclusion period, giving all dates an equal chance of selection.
**Mkoma (2024) (33)**	Outcome: The primary outcome was hospitalisation due to COVID-19, defined as cases where COVID-19 was recorded as the main reason for admission. This was identified using ICD-10 codes B34.2, B34.2A, B97.2, or B97.2A.<br>Population:The study included all Danish residents aged 18 or older who tested positive for SARS-CoV-2 for the first time between January 1, 2020, and August 31, 2022. To explore symptom patterns more comprehensively, the researchers also assessed symptom reporting in three time windows relative to the COVID-19 diagnosis: Within 6 months before diagnosis; From 0 to 4 weeks after diagnosis; From >4 weeks to 6 months after diagnosis.
**Nersesjan (2023) (40)**	Outcome: The primary outcomes were the first diagnosis of any mental disorder (ICD-10: F00–F99) and the first redemption of psychotropic medications (ATC: N05–N06). Secondary outcomes included specific psychiatric diagnoses—organic mental disorders (F00–F09), schizophrenia spectrum disorders (F20–F29), mood disorders (F30–F39), and stress-related or somatoform disorders (F40–F48)—as well as specific medication groups: antidepressants

	(N06A), antipsychotics (N05A), anxiolytics (N05B), and antidementia drugs (N06D). The timing of onset was based on the first psychiatric contact or the first time a relevant prescription was filled.<br>Population: The study included all individuals in Denmark with PCR test records for SARS-CoV-2 from the national microbiology registry. Each person's test history was used to assign them to one of three mutually exclusive and time-varying exposure groups: Not tested (until their first test), Tested negative, and Tested positive (starting from the first positive test and remaining there thereafter).
**Reme (2023) (34)**	Outcome: The primary outcome was the presence of post-COVID condition, as recorded by general practitioners in primary or emergency care using ICPC-2 coding. Specifically, this required the use of the R992 COVID-19 code alongside at least one additional code indicating a persistent symptom (e.g., fatigue or pain). The analysis focused on symptoms documented 90 to 180 days after the first positive SARS-CoV-2 test, aligning with the WHO definition of post-COVID condition—persistent symptoms three months after infection.<br>Population: The study included all Norwegian residents aged 30 to 70 years (working-age individuals) as of January 1, 2020, who had their first PCR-confirmed SARS-CoV-2 infection between July 1, 2020, and January 23, 2022. Only individuals with no prior COVID-19 infection were included, ensuring any post-COVID symptoms were not due to earlier infections.
**Rømer (2023) (39)**	Outcome: The primary outcome was psychiatric hospital admission, defined as psychiatric stays longer than 24 hours beginning at least one day after study inclusion, with ICD-10 codes F20–F50 (affective, anxiety, and psychotic disorders) as the primary diagnosis. Transfers from somatic to psychiatric units were also included. Diagnoses such as personality disorders, ADHD, and intellectual disability were excluded due to unlikely causal links with COVID-19. The secondary outcome was the first-time prescription of any psychoactive medication, regardless of clinical indication, due to limited data on prescribing rationale.<br>Population: All adults (aged 18 and older) residing in Denmark (excluding Greenland and the Faroe Islands) as of January 1, 2020, were eligible. Individuals were only excluded if they had invalid SARS-CoV-2 PCR test dates (e.g., tests recorded posthumously). Importantly, people with ongoing psychiatric hospitalizations at baseline were included. SARS-CoV-2 infection was defined solely by the registration of a positive PCR test, without consideration of symptom severity or hospitalization status.
**Skei (2023) (28)**	Outcome: The main outcome was work status at 6 months, 1 year, and 2 years following discharge from a sepsis-related hospitalization. Work status was classified into four categories: Returned to work (RTW): No sickness or medical benefits at the time point; Ever RTW: Initially returned to work but later received benefits again; Never RTW: Received sickness or medical benefits continuously across all follow-up points; Deceased.<br>Secondary outcomes included: Trends in return to work across the full study period; Sustainable RTW, defined as having no sickness or medical benefit for at least 31 consecutive days post-discharge.<br>Population: The study included patients aged 18–60 years hospitalized with sepsis in Norwegian public hospitals between

	2010 and 2021. Sepsis was defined using ICD-10 codes indicating either an infection with accompanying organ dysfunction (implicit sepsis) or a specific sepsis diagnosis (explicit sepsis).<br>Patients were excluded if they had: Received a disability pension prior to the sepsis admission; Died before hospital discharge.<br>The upper age limit ensured the analysis focused on working-age individuals and excluded retirement-related work absence.
**Skyrud (2021) (18)**	Outcome: The study examined all-cause use of primary and specialist health care services from 1 to 24 weeks (approximately 1 to 6 months) after the week of SARS-CoV-2 testing.<br>Two weekly binary outcomes were defined: Whether the person had at least one primary care contact (general practitioner or emergency ward); Whether the person had at least one specialist care contact (hospital outpatient or inpatient services).<br>If an increase in health care use was observed following mild COVID-19, the study further explored potential underlying reasons using ICPC-2 diagnostic codes.<br>Population: The population included all adults (≥20 years) living in Norway as of January 1, 2020 (including those born in 2020), who underwent PCR testing for SARS-CoV-2 between March 1, 2020 and February 1, 2021. Individuals were followed for at least 2 months prior to and 3 months after the test date (most for up to 6 months); Non-residents (e.g., tourists) were excluded. Two groups were defined: No COVID-19: Individuals with a negative PCR test; Mild COVID-19: Individuals with a positive PCR test who were not hospitalized.
**Skyrud (2021) (26)**	Outcome: The study assessed doctor-certified sick leave (for any cause) before and after SARS-CoV-2 testing, using a panel data structure and a difference-in-differences approach.<br>Sick leave patterns were compared between: Individuals with a negative test, Individuals with a positive test but not hospitalized (mild COVID-19), Individuals with a positive test and hospitalized (severe COVID-19), with analyses stratified by age and sex.<br>Population: The population included all Norwegian residents aged 20–70 years with an employment contract who were PCR-tested for SARS-CoV-2 between March 1, 2020, and February 1, 2021 (N = 1,177,274; mean age 40 years; 46% men).
**Skyrud (2022) (19)**	Outcome: The study examined physiotherapy consultations in primary care/community services, tracking visits from 12 weeks before hospital admission to 36 weeks after hospital discharge for patients hospitalized with either COVID-19 or other respiratory tract infections (RTIs).<br>Population: The population included adults aged 18–80 years who were hospitalized in Norway between July 1, 2017, and August 1, 2021, and who could be observed for at least 24 weeks before and after hospitalization.<br>Patients aged 80+ and non-residents (e.g., tourists, temporary workers) were excluded. Hospitalized individuals were classified into two mutually exclusive diagnosis groups based on NPR records: COVID-19 (ICD-10: U071, U072); Other RTIs (e.g., influenza).
**Spetz (2024) (29)**	Outcome: The primary outcome was COVID-19-related sick leave, defined as the initiation of a sickness benefit episode (≥15 days of sick leave) with COVID-19 registered as the cause. This had to

	occur within a time window of 7 days before to 30 days after an individual's first positive SARS-CoV-2 PCR test.<br>Population:The study included all gainfully employed individuals aged 18–64 years, residing in Sweden on January 1, 2020, who had a first positive SARS-CoV-2 PCR test between January 1, 2020, and August 31, 2021 (n = 661,780). Only sick leave episodes ≥15 days, for which sickness benefits were paid by the Swedish Social Insurance Agency (SSIA), were captured. Shorter sick leave episodes (<15 days), as well as those among self-employed individuals, students, and the unemployed, were not included.

Supplementary Table 3.

Search strategy

MEDLINE (Ovid) ALL 1946 May 30 & Embase 1974 to 2024 May 30

1	((chronic* or long or linger* or longterm or long-term or persist* or post acute or postacute or sustain*) adj covid*).ti,ab,kf.
2	(Post covid* adj3 (illness* or syndrome* or symptom* or condition* or complain* or fatigue or impair* or complication* or pain)).ti,ab,kf.
3	(Prolonged adj3 covid*).ti,ab,kf.
4	Post-Acute COVID-19 Syndrome/
5	or/1-4
6	((chronic or linger* or long-term or longterm or persist* or prolong* or sustain*) adj3 (complication* or infect* or symptom* or syndrome* or burden* or consequence* or outcome* or complain* or impair* or fatigue or pain or effect or effects)).ti,ab,kf.
7	(Long-haul* or longhaul*).ti,ab,kf.
8	(Prolonged adj3 recovery).ti,ab,kf.
9	sequelae*.ti,ab,kf.
10	(sick* or sickleave* or absence* or leave* or sick* or primary* or specialist* or consultation* or healthcare* or visit*).ti,ab,kf.
11	or/6-10
12	exp Coronavirus/ or exp Coronavirus Infections/
13	(coronavirus* or corona virus* or covid* or sars-cov* or sarscov* or omicron* or omikron*).mp.
14	(2019-ncov or ncov19 or ncov-19 or 2019-novel CoV or sars-cov2 or sars-cov-2 or sarscov2 or sarscov-2 or Sars-coronavirus2 or Sars-coronavirus-2 or ((novel or new or nouveau) adj2 (CoV or nCoV or Pandemi*))).mp.
15	COVID-19.px,ox. or severe acute respiratory syndrome coronavirus 2.os.

16	or/12-15
17	11 and 16
18	5 or 17
19	"Scandinavian and Nordic Countries"/ or Denmark/ or Greenland/ or Finland/ or Iceland/ or Norway/ or Svalbard/ or Sweden/
20	Arctic Regions/
21	(arctic or subarctic or scandinavia* or nordic countr* or denmark or faroe islands or danish or greenland* or finland or lapland or lappland or finnish or iceland or icelandic or norway or norwegian or svalbard or sweden or swedish or saami or sapmi).ti,ab,kf.
22	or/19-21
23	18 and 22
24	(covid and (senfolger or senfoelger or ettervirkninger or senvirkninger)).ot.
25	23 or 24
26	limit 25 to yr="2020 -Current"

Webpages reviewed as part of the grey literature searches:
Åland: ahs.ax
Denmark: ssi.dk, sst.dk/da/, dst.dk
Finland: stm.fi, stat.fi, thl.fi
Færøyene: hfs.fo
Greenland: peqqik.gl
Iceland: statice.is, government.is, island.is
Norway: fhi.no, nav.no, ssb.no
Sweden: folkhalsomyndigheten.se, forsakringskassan.se, scb.se
